# Current Understanding of the Immune Response after COVID-19 Vaccination

**DOI:** 10.3390/vaccines12030250

**Published:** 2024-02-28

**Authors:** Shunbin Ning, Davide Firinu

**Affiliations:** 1 Department of Medicine, Center of Excellence for Inflammation, Infectious Diseases, and Immunity, Quillen College of Medicine, East Tennessee State University, Johnson City, TN 37614, USA; 2 Department of Medical Sciences and Public Health, University of Cagliari, 09124 Cagliari, Italy

The global vaccination campaign against SARS-CoV-2, the virus responsible for COVID-19, has been a monumental endeavor, marked by unprecedented collaboration between scientific researchers and pharmaceutical companies. One of the most promising tools in this fight is vaccination. As billions around the world receive COVID-19 vaccines, understanding the immune response elicited by these vaccines is crucial for optimizing public health strategies and refining vaccine development. In this Special Issue, thirteen publications shed light on the evolving landscape of immunity post-COVID-19 vaccination.

COVID-19 vaccines primarily aim to induce an immune response that protects against severe illness and reduces the transmission of the virus. A single vaccine dose may suffice for previously infected individuals. It takes at least a couple of weeks for the body to mount an effective immune response after any COVID-19 vaccination. Most vaccines achieve this by presenting a harmless part of the virus, often the spike protein, to the immune system. This presentation stimulates the production of antibodies and activates T cells. T cells can recognize and destroy cells infected with the virus. COVID-19 vaccines, particularly mRNA vaccines, have been shown to elicit strong T-cell responses [1,2]. CD4+ T lymphocytes, also known as helper T cells, remained at significantly higher levels six months after vaccination with mRNA-based COVID-19 vaccines [3]. This cellular immunity is vital for long-term protection and may contribute to preventing severe disease, even if antibody levels wane over time.

Understanding the duration of immunity conferred by COVID-19 vaccines is essential for determining the necessity of booster shots. Factors such as the emergence of SARS-CoV-2 variants have raised questions about the effectiveness of existing vaccines. Studies suggest that vaccines, particularly mRNA vaccines, remain effective against severe disease caused by variants, although there may be a modest reduction in efficacy against some variants [4]. Continuous surveillance and research are crucial for adapting vaccines to new variants and optimizing their efficacy.

COVID-19 vaccines may also provide a certain level of cross-protection against related coronaviruses [5]. Additionally, studies are exploring the feasibility and efficacy of heterologous vaccination, where different types of vaccines are administered in a mixed-dose schedule [6]. These approaches could offer flexibility in vaccination campaigns and address supply chain challenges.

While some regions have made significant progress in vaccination efforts, global disparities persist. Access to vaccines, especially in low-income countries, remains a critical challenge. Addressing these disparities is not only an ethical imperative but also essential for achieving global herd immunity and preventing the emergence of new variants.

Ensuring the safety of COVID-19 vaccines is paramount for building public trust and achieving widespread vaccination. Monitoring systems, such as the Vaccine Adverse Event Reporting System (VAERS), play a crucial role in assessing and addressing potential side effects. The overwhelming evidence supports the safety of COVID-19 vaccines, with reported side effects generally mild and temporary.

In conclusion, the current understanding of the immune response after COVID-19 vaccination provides a foundation for shaping future vaccine development, booster strategies, and global vaccination campaigns. As we navigate these uncertainties, continued collaboration among scientists, policymakers, and the public is essential to overcoming the COVID-19 pandemic and preparing for future challenges in global health.
